# Supplementary material for: TED—Trazodone Efficacy in Depression: A Naturalistic Study on the Efficacy of Trazodone in an Extended-Release Formulation Compared to SSRIs in Patients with a Depressive Episode—Preliminary Report
Source: Brain Sci. 2023 Jan 2;13(1):86. doi: 10.3390/brainsci13010086 (PMC9856641; doi:10.3390/brainsci13010086)
Supplement: Supplementary file 1 [file brainsci-13-00086-s001.zip › brainsci-2084760-supplementary.pdf]

Figure S1. Line graphs for each outcome measure with mean values represented on Y-axis and sequential time points on X-axis, with separate lines for each treatment option (SSRI or trazodone). (**a**) Montgomery-Åsberg Depression Rating Scale (MADRS); (**b**) Quick Inventory of Depressive Symptomatology - clinician rating (QIDS); (**c**) Quick Inventory of Depressive Symptomatology – self-rating (QIDS-SR) (**d**) Hamilton Anxiety Rating Scale (HAM-A); (e) Athens Insomnia Scale (AIS; (**f**) Sheehan Disability Scale (SDS); (**g**) Snaith-Hamilton Pleasure Scale (SHAPS). * = p ≤ 0.05, ** = p ≤ 0.01, *** = p ≤0.001.

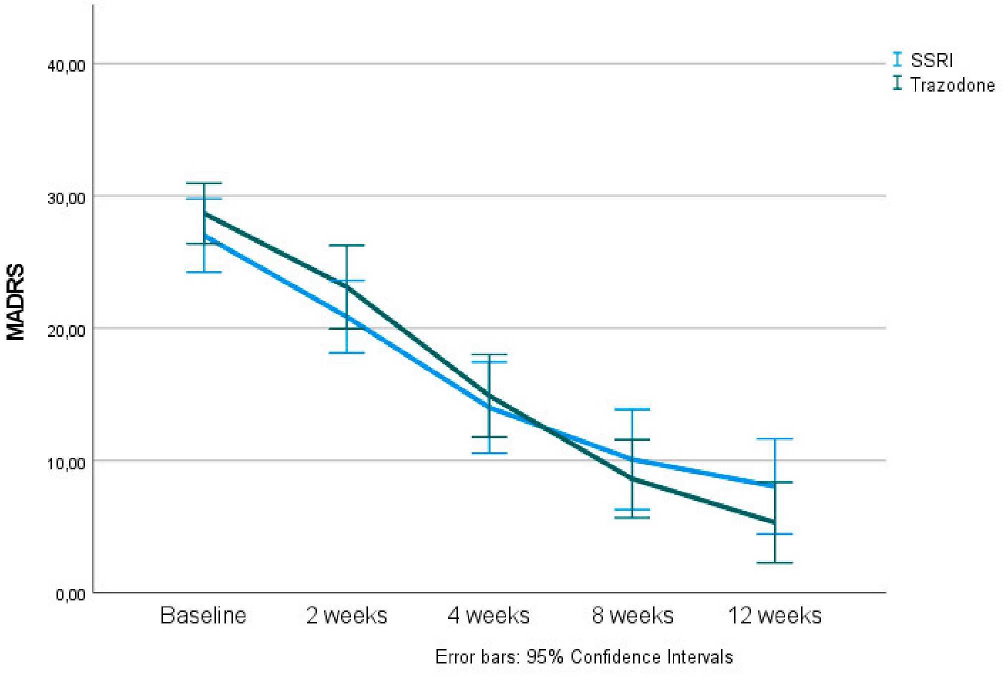

(**a**)

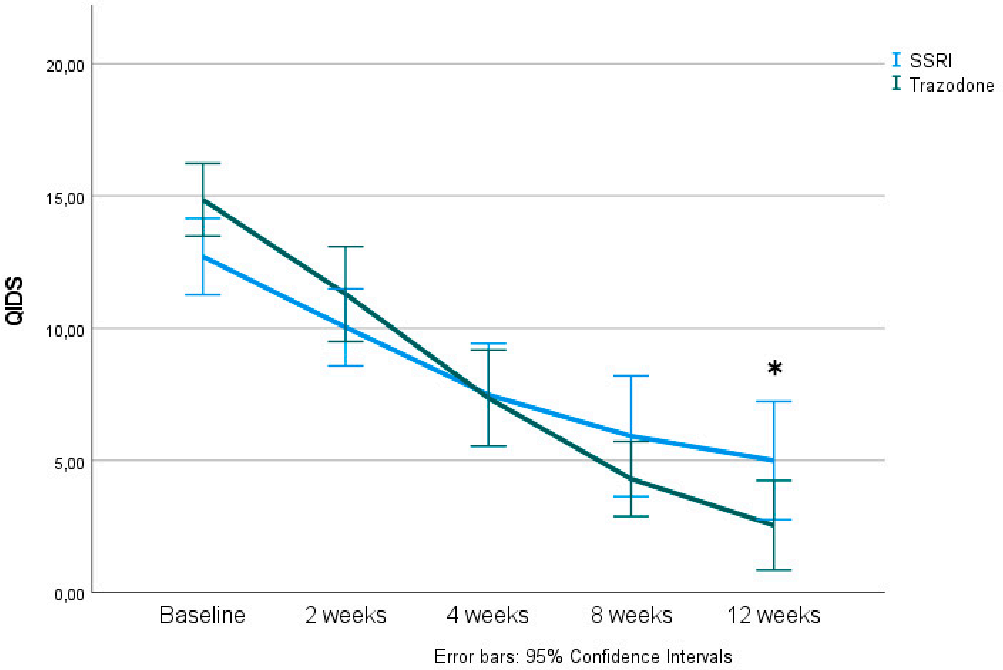
SSRI
Trazodone
20,00
15,00
10,00
5,00
0,00
QIDS
*
Baseline
2 weeks
4 weeks
8 weeks
12 weeks
Error bars: 95% Confidence Intervals

(b)

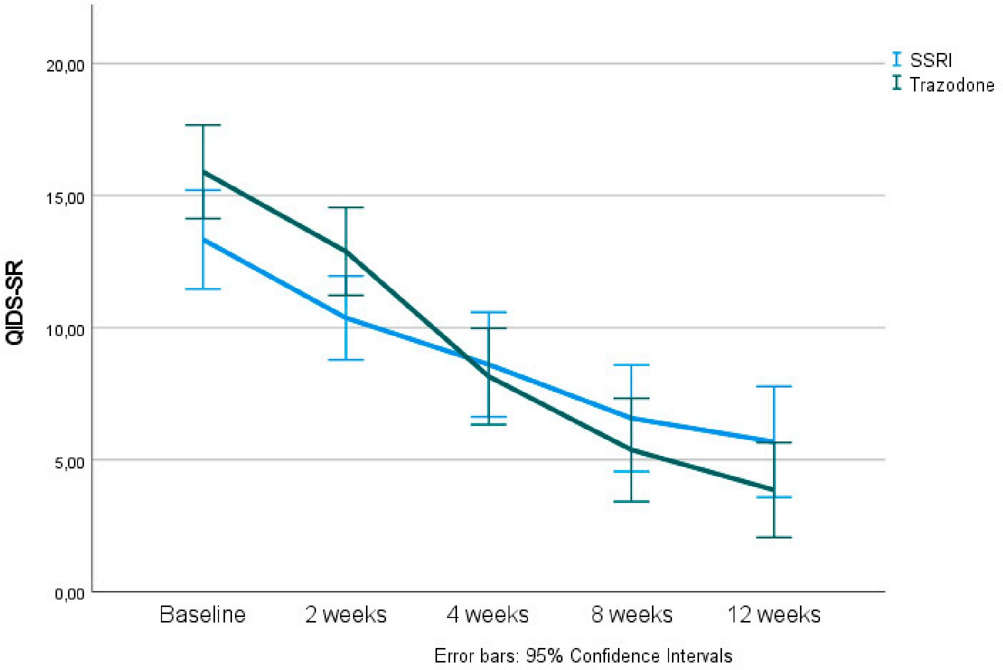
SSRI
Trazodone
20,00
15,00
10,00
5,00
0,00
QIDS-SR
Baseline
2 weeks
4 weeks
8 weeks
12 weeks
Error bars: 95% Confidence Intervals

(c)

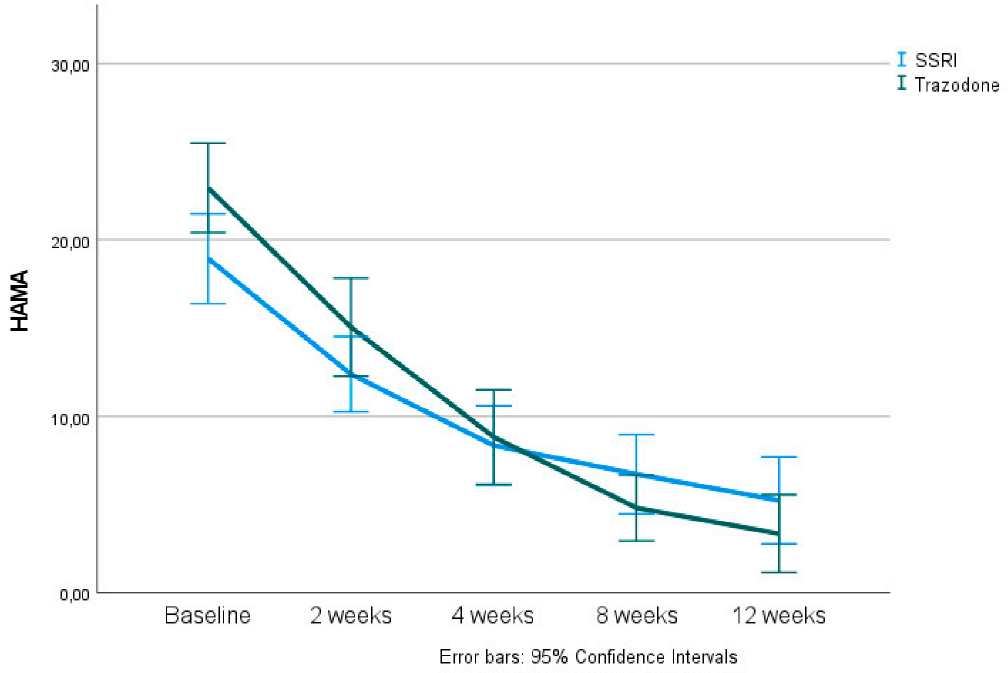
SSRI
Trazodone
HAMA
30,00
20,00
10,00
0,00
Baseline
2 weeks
4 weeks
8 weeks
12 weeks
Error bars: 95% Confidence Intervals

(d)

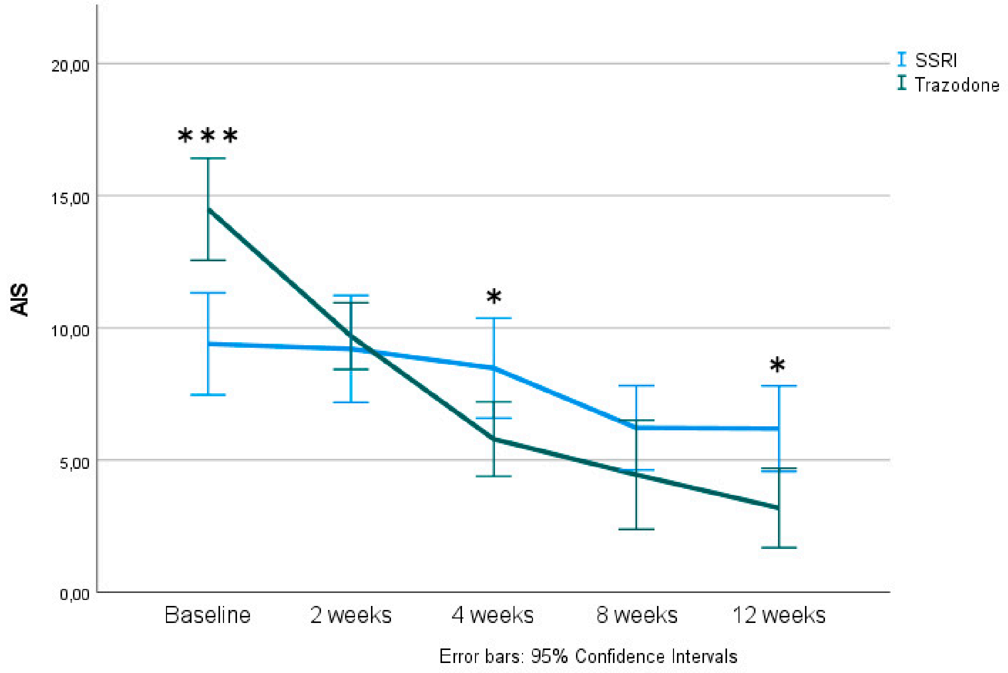
SSRI
Trazodone
AIS
20,00
15,00
10,00
5,00
0,00
***
*
*
Baseline
2 weeks
4 weeks
8 weeks
12 weeks
Error bars: 95% Confidence Intervals

(e)

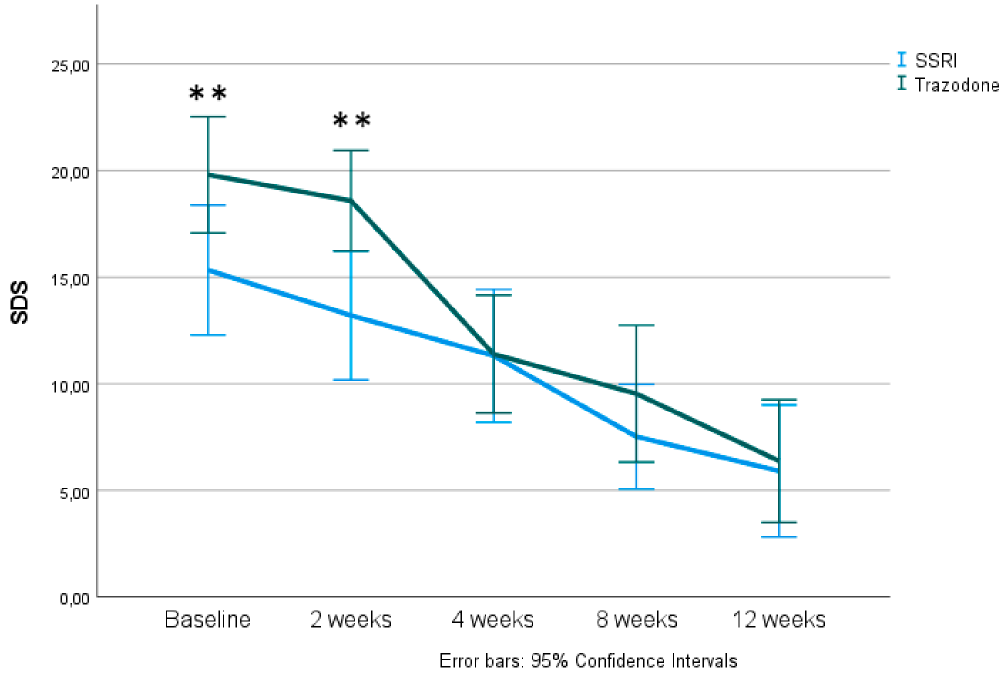
SSRI
Trazodone
25,00
20,00
15,00
10,00
5,00
0,00
SDS
**
**
Baseline
2 weeks
4 weeks
8 weeks
12 weeks
Error bars: 95% Confidence Intervals

(f)

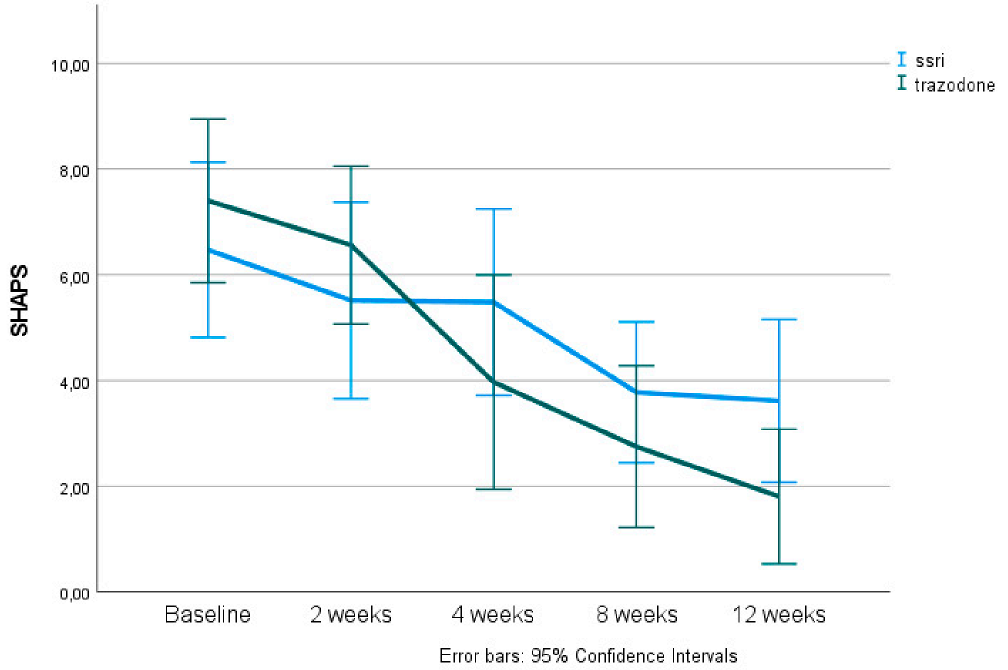
ssri
trazodone
10,00
8,00
6,00
4,00
2,00
0,00
SHAPS
Baseline
2 weeks
4 weeks
8 weeks
12 weeks
Error bars: 95% Confidence Intervals

(g)
